# Non-Conforming Still’s Disease With Low Ferritin and No Skin Rash: A Case Report

**DOI:** 10.7759/cureus.9168

**Published:** 2020-07-13

**Authors:** Dawlat Khan, Muhammad Umar Saddique, Muhammad B Jamshaid, Zohaib Yousaf, Mohamed A Yassin

**Affiliations:** 1 Internal Medicine, Hamad Medical Corporation, Doha, QAT; 2 Internal Medicine, Hamad General Hospital, Doha, QAT; 3 Clinical Research, Dresden International University, Dresden, DEU; 4 Hematology and Oncology, Hamad General Hospital, Doha, QAT

**Keywords:** still disease, high ferritin, fuo, puo

## Abstract

Adult Still's disease (ASD) is an inflammatory disorder with an unclear etiology. It is a rare disease that was first described more than a century ago. Its common characteristics are daily fevers, arthritis, and skin rash. ASD is diagnosed after excluding infections, malignancies, and connective tissue diseases. It has a female predominance. Classic skin rash and high serum ferritin levels are commonly associated with this condition and help in the diagnosis. Due to a lack of pathognomic clinical and laboratory features, a valid diagnostic criterion, the Yamaguchi criteria, is generally used for the diagnosis. The disease has a good prognosis with appropriate treatment.

We present the case of a young gentleman who presented with fever, anemia, and leucocytosis; however, his serum ferritin levels were normal, and there was no typical salmon-colored skin rash. Hyperferritinemia developed later in the disease course, leading to a diagnosis of ASD.

## Introduction

Adult Still's disease (ASD) is a rare chronic systemic inflammatory disorder. Its annual incidence is 0.16 cases per 100,000 people, with a female-to-male ratio of 3:2 [1,2]. Initially described in children by George F. Still, in 1897, it remained overlooked until it was discussed again by Bywaters in 1971 [3]. It has a bimodal distribution with peaks in the age groups of 15-25 years and 36-46 years. The proposed etiology is multifactorial involving genetic factors, human leukocyte antigen (HLA), with possible infections and malignancies acting as triggers [4]. The classical presentation includes daily recurring fever (quotidian fever) or double quotidian fever (two fever spikes a day), accompanied by a salmon-pink maculopapular eruption overlapping with fever. Other signs that have been reported include arthralgia or arthritis, sore throat, weight loss, lymphadenopathy, and hepatosplenomegaly. Hyperferritinemia is very common, but its absence does not exclude ASD. It is a diagnosis of exclusion. Diagnosis is generally made after ruling out infections, malignancies, and connective tissue diseases by using the Yamaguchi or Fautrel criteria [5]. The prognosis is usually favorable with timely and appropriate therapy. In this report, we discuss an unusual case of ASD in a patient who presented without a skin rash and showed a delayed presentation of hyperferritinemia.

## Case presentation

An 18-year-old, previously healthy gentleman presented with a three-week history of high-grade fever, dry cough, throat pain, and arthralgia involving knees, ankles, and elbows. The fever was intermittent, with one episode per day and the temperature rising as high as 39 °C, which was relieved by acetaminophen. He had a history of unintentional weight loss and fatigue. Before presenting to the hospital, he had received a seven-day course of empiric antibiotics for suspected bacterial pharyngitis with no improvement. He denied any cough, hemoptysis, night sweats, rash, and gastrointestinal or urinary symptoms. He also denied any history of sick contacts, close tuberculosis (TB) contacts, and recent travel. There was no history of recurrent fever or joint pains in his family.

Clinical examination revealed a conscious, oriented, and hemodynamically stable patient with a temperature of 39.2 °C. He had mild pallor but not icteric, and small painless, soft, and mobile cervical lymph nodes were palpable. The rest of the examination including ankle and knee joints were unremarkable with no signs of inflammation.

He had microcytic anemia, marked neutrophilic predominant leukocytosis, high C-reactive protein (CRP), mild transaminitis, and normal lactate level. The blood and urine cultures taken during fever spikes were sterile. He had a negative malaria test and negative serology for brucellosis and mycoplasma. Additionally, viral hepatitis serology and respiratory viral panel, cytomegalovirus (CMV), Epstein-Barr virus (EBV), and adenoviral polymerase chain reaction (PCR) were all negative, He also had a twice-negative sputum acid-fast bacillus (AFB) stain and sputum TB PCR. The autoimmune workup was also normal (Tables 1, 2).

**Table 1:** Hematological and biochemical investigations ALT: alanine transaminase; AST: aminotransferase; LDH: lactate dehydrogenase; CRP: C-reactive protein; TIBC: total iron-binding capacity

Lab parameters	At admission	Fourth week	Eighth week	Reference range
Hemoglobin	10.4	10.7	12.4	13-17
White blood cell count	28,000	25,000	10.5	4-10 x10^3^/µL
Neutrophils	87%	81%	40%	40-60%
Platelets	459	367	300	150-400 x10^3^/µL
ALT	89	53	13	0-40 U/L
AST	62	51	20	0-37 U/L
Alkaline phosphatase­­­­­­­­­­­	104	110	86	40-120 U/L
Total bilirubin	5	---	12	4-24 mmol/L
Creatinine	53	63	70	70-115 mmol/L
Sodium	135	141	143	135-145 mmol/L
Haptoglobin	657	---	---	30-200 mg/dl
LDH	328	---	---	105-235 U/L
CRP	279	150	14	0-5 mg/L
Procalcitonin	0.72	---	---	0.5 ng/mL
Serum iron	7.50	---	7.50	4.80-24.7 mmol/L
Serum ferritin	35	>7,000	601	30-400 mcg/L
TIBC	45	--	40	40-80 mmol/L
Lactic acid	1.1	--	---	0.5-1.6 mmol/L
Triglyceride	2.2	---	0.79	<1.7 mmol/L

**Table 2:** Bacteriology and autoimmune workup AFB: acid-fast bacillus; TB: tuberculosis; PCR: polymerase chain reaction; HIV: human immunodeficiency virus; ELISA: enzyme-linked immunosorbent assay; CMV: cytomegalovirus; EBV: Epstein-Barr virus; Anti-CCP: anti-cyclic citrullinated peptide; ANA: antinuclear antibody; Anti-dsDNA: anti-double-stranded DNA; ANCA: antineutrophil cytoplasmic antibodies

Test	Result
Sputum AFB and TB-PCR (two sets)	Negative
QuantiFERON TB	Negative
Brucellosis (IgM/IgG)	Negative
Mycoplasma pneumoniae serology	Negative
Blood and urine culture	No growth
Hepatitis B and C	Negative
HIV (ELISA)	Negative
Blood CMV/EBV/adenoviral PCR	Negative
Rheumatoid factor	Normal
Anti-CCP	Negative
ANA	Negative
Anti-dsDNA	Negative
ANCA	Negative
Anticardiolipin	Negative

Electrocardiogram (ECG) and chest X-ray were unremarkable. Ultrasound abdomen was negative for hepatosplenomegaly, ascites, or fluid collection in the abdomen and pelvis. Echocardiography did not show any infective endocarditis. CT of the chest, abdomen, and pelvis with contrast revealed no enlarged lymph nodes or collections. Peripheral blood film did not reveal any blast cells, and bone marrow aspiration and biopsy showed healthy cellular bone marrow with trilineage hematopoiesis and no hemophagocytosis.

**Figure 1:**
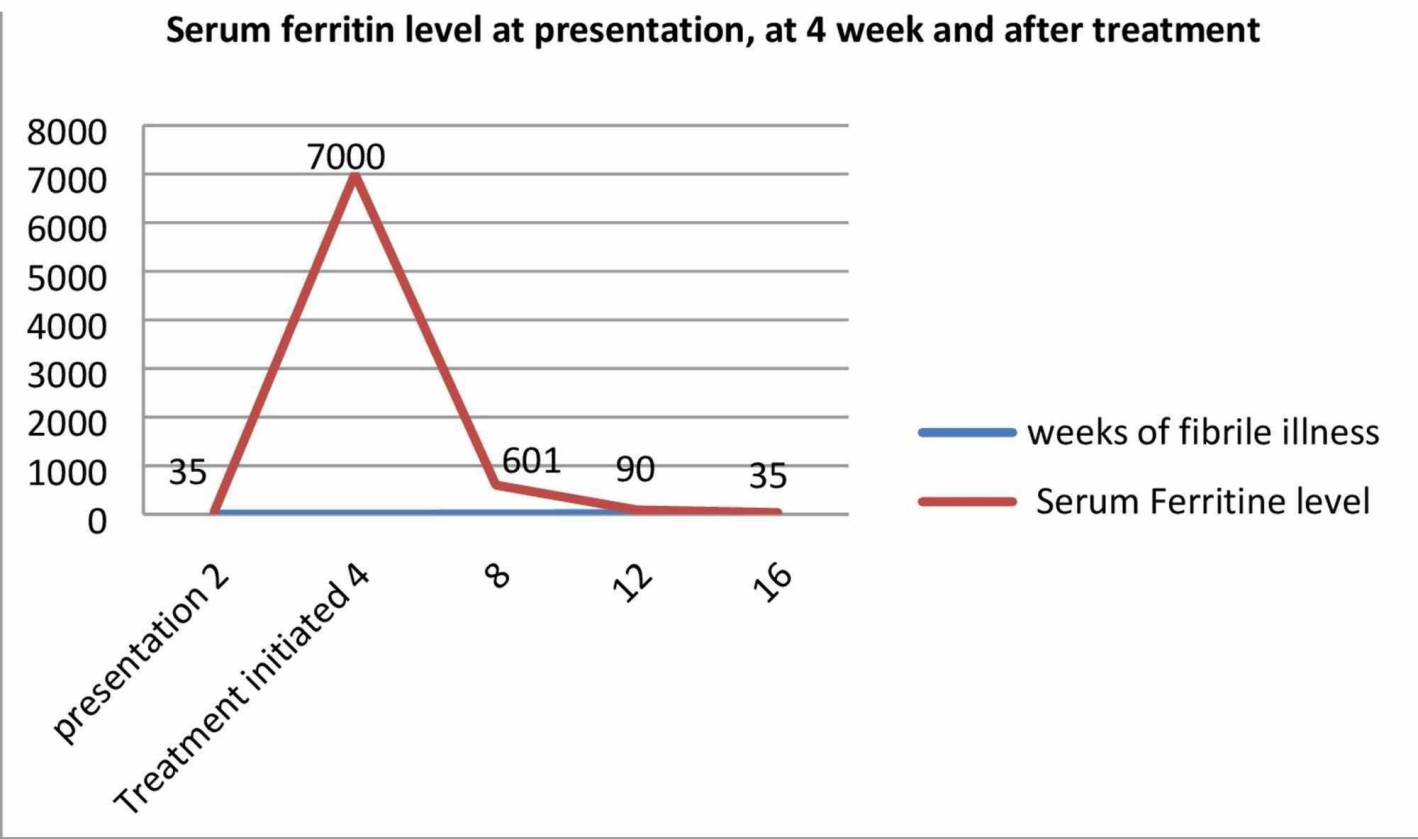
Serum ferritin levels at presentation, at four weeks, and after therapy

The initial impression was of bacterial infection, and the patient received broad-spectrum antibiotics for the appropriate duration; however, he continued to spike daily fever. He had an unremarkable ferritin level at presentation. After the initial workup virtually excluded TB, ongoing occult infection, lymphoma, and hematologic malignancy, ferritin was repeated. It was found that the patient exhibited marked hyperferritinemia (Figure 1).

After excluding all plausible alternative diagnoses, ASD was diagnosed based on the Yamaguchi's criteria. The patient was treated with oral steroids. Consequently, his fever subsided, and he started gaining weight. Arthralgia subsided more gradually over time. Prednisolone was tapered down, and methotrexate was added to the treatment regimen. The patient responded well to the treatment. Subsequently, all his symptoms improved and serum ferritin levels normalized, and he has been symptom-free for almost a year now.

## Discussion

The diagnosis of ASD is often delayed as it is a diagnosis of exclusion. The etiology and pathogenesis are still unclear, but some bacterial and viral infections and certain malignancies may act as inciting factors. No clear association between specific HLA loci or single genes and ASD has been established so far [6]. Specific cytokines may be involved in the pathogenesis of ASD. Serum levels of interleukin 6 (IL-6) and IL-18 have been found to exhibit a correlation with the clinical activity score of ASD [7].

Pyrexia of unknown origin (PUO) refers to a prolonged febrile illness without an established etiology despite extensive evaluation and diagnostic testing. Infections, non-infectious inflammatory etiology, and malignancies are common causes, and a significant number of patients remain undiagnosed [8]. ASD is the most common rheumatologic disorder presenting as PUO in young and middle-aged adults [9].

The most common symptoms of ASD are high-grade fever (39 °C or above) in more than 95% of patients with one or more daily fever spikes. Also, large joint polyarthritis or arthralgia is also present in about 95% of cases as well as worsening joint pain during fever. The fusion of wrist joints is one of the specific radiological presentations [10]. The typical skin rash that appears in 80% of patients is salmon-colored, non-pruritic, non-scarring, and grows during fever spikes with positive Koebner phenomenon. Nonsuppurative pharyngitis, hepatomegaly, transaminitis, serositis, and rare involvement of renal, ophthalmic, and neurological systems have also been reported [11].

The disease presentation may be monophasic, intermittent, or chronic articular-type; each type roughly accounts for one-third of the cases. The type of presentation has an impact on prognosis [12]. Due to a lack of pathognomic clinical features and definitive diagnostic laboratory tests, ASD is usually diagnosed by applying the widely accepted Yamaguchi criteria. The presence of five features, with at least two of them being major criteria, is required for the diagnosis (Table 3) [13]. The Fautrel criteria have a higher specificity but lower sensitivity when compared to the Yamaguchi criteria; therefore, the latter is used more frequently to rule out ASD [14].

**Table 3:** Yamaguchi criteria for the diagnosis of adult Still's disease Diagnosis requires the patients to have at least five features, with at least two of them being major diagnostic criteria ANA: antinuclear antibody; LFT: liver function test

Major criteria	Minor criteria
Fever of at least 39 °C for ≥1 week	Sore throat
Arthralgia for ≥2 weeks	Lymphadenopathy
Evanescent rash during fever spike	Hepatomegaly or splenomegaly
Leukocytosis (>10,000) with neutrophilia (>80%)	Negative for ANA and rheumatoid factor
	Abnormal LFTs

The characteristic laboratory findings are lymphocytosis with neutrophilia, normocytic normochromic anemia and thrombocytosis, high CRP, erythrocyte sedimentation rate (ESR), and transaminitis [15]. Marked hyperferritinemia is a common feature of ASD but is not pathognomonic. Elevated ferritin levels are associated with other rheumatological conditions. ASD is associated with a higher serum ferritin level of more than five times the upper limits of normal (>1,000 ug/dl), sometimes ranging up to >50,000 ug/dl [16]. Ferritin level also correlates with disease activity and is used as a biomarker to monitor therapeutic response [17]. The hypothesized mechanism involves cytokines-mediated ferritin synthesis. However, cases of ASD without a high ferritin level have been reported [18].

In our case, the serum ferritin level was lagging at the time of disease onset, which kept the suspicion of ASD low. The glycosylated ferritin level in ASD patients is lower (<20%) than that of an average healthy person (50-80%). The sensitivity and specificity of <20% glycosylated ferritin in ASD are 78 and 64% respectively. A combination of the rise in serum ferritin and glycosylated ferritin levels has less sensitivity (43%) but high specificity (93%) for ASD [16].

The common treatment options available for ASD are non-steroidal anti-inflammatory drugs (NSAIDs), glucocorticoids, and disease-modifying anti-rheumatic drugs (DMARDs). The preferred treatment option for the articular disease is methotrexate; however, anakinra is preferred for systemic disease. Observational studies have suggested that DMARDs started within six months of the onset of chronic articular involvement may be associated with lower rates of joint injury, but prospective studies are needed to validate these observations [19]. Anti-IL-1 based therapies help to prevent the progression of amyloid [20]. Our patient had a moderate disease presentation. He was initially treated with oral steroids and later switched to methotrexate due to weight gain and persistent articular symptoms. The most severe complication of ASD is hemophagocytic lymphohistiocytosis (HLH), a syndrome resulting from excessive immune system activation.

## Conclusions

ASD is a difficult disease to diagnose. It should be a significant consideration as a cause of PUO in young and middle-aged patients when rheumatologic, infectious, and malignant workups are negative. Atypical presentations may involve the absence of the typical skin rash and low serum ferritin levels in early disease. This phenomenon is rare and would be a diagnostic dilemma for the clinician, and may lead to underdiagnosis. The authors recommend that serum ferritin levels be repeated if PUO workup is unyielding despite an initial normal level. Early diagnosis and initiation of appropriate therapy can help to prevent complications and improve the prognosis for patients with ASD.

## References

[1] Magadur-Joly G, Billaud E, Barrier JH, Pennec YL, Masson C, Renou P, Prost A (1995). Epidemiology of adult Still's disease: estimate of the incidence by a retrospective study in west France. Ann Rheum Dis.

[2] Balci MA, Pamuk ÖN, Pamuk GE, Uzundere FK, Donmez S (2015). Epidemiology and outcome of adult-onset Still's disease in Northwestern Thrace region in Turkey. Clin Exp Rheumatol.

[3] Bywaters EG (1971). Still's disease in the adult. Ann Rheum Dis.

[4] Colebunders R, Stevens WJ, Vanagt E, Snoeck J (1984). Adult Still's disease caused by Yersinia enterocolitica infection. Arch Intern Med.

[5] Maset M, Costa EG, Carniello GS, Grazioli S, Ghersetti M, Casarin P (2016). Fever and erythema: exclude all and then… think of Still’s disease!. Italian J Med.

[6] Siddiqui M, Putman MS, Dua AB (2016). Adult-onset Still’s disease: current challenges and future prospects. Open Access Rheumatol.

[7] Chen DY, Lan JL, Lin FJ, Hsieh TY (2004). Proinflammatory cytokine profiles in sera and pathological tissues of patients with active untreated adult onset Still's disease. J Rheumatol.

[8] Efstathiou SP, Pefanis AV, Tsiakou AG, Skeva II, Tsioulos DI, Achimastos AD, Mountokalakis TD (2010). Fever of unknown origin: discrimination between infectious and non-infectious causes. Eur J Intern Med.

[9] Arnow PM, Flaherty JP (1997). Fever of unknown origin. Lancet.

[10] Björkengren AG, Pathria MN, Sartoris DJ, Terkeltaub R, Esdaile JM, Weisman M, Resnick D (1987). Carpal alterations in adult-onset Still disease, juvenile chronic arthritis, and adult-onset rheumatoid arthritis: comparative study. Radiology.

[11] Cozzi A, Papagrigoraki A, Biasi D, Colato C, Girolomoni G (2016). Cutaneous manifestations of adult-onset Still’s disease: a case report and review of literature. Clin Rheumatol.

[12] Yerra S, Tlhabano L, Vasamsetty T (2016). Case report of atypical Still’s disease: a diagnosis of exclusion. Int Med Case Rep J.

[13] Yamaguchi M, Ohta A, Tsunematsu T (1992). Preliminary criteria for classification of adult Still's disease. J Rheumatol.

[14] Fautrel B, Zing E, Golmard JL (2002). Proposal for a new set of classification criteria for adult-onset Still disease. Medicine (Baltimore).

[15] Kontzias A, Efthimiou P (2008). Adult-onset Still's disease: pathogenesis, clinical manifestations and therapeutic advances. Drugs.

[16] Mehta B, Efthimiou P (2012). Ferritin in adult-onset Still’s disease: just a useful innocent bystander?. Int J Inflam.

[17] Kirino Y, Kawaguchi Y, Tada Y (2018). Beneficial use of serum ferritin and heme oxygenase-1 as biomarkers in adult-onset Still’s disease: a multicenter retrospective study. Mod Rheumatol.

[18] Omagari K, Matsunaga Y, Yamashita H (2003). Successful treatment with cyclosporin in adult-onset Still disease manifesting as acute hepatitis with marked hyperferritinemia. Am J Med Sci.

[19] Franchini S, Dagna L, Salvo F, Aiello P, Baldissera E, Sabbadini MG (2010). Efficacy of traditional and biologic agents in different clinical phenotypes of adult‐onset Still's disease. Arthritis Rheum.

[20] Topaloglu R, Batu ED, Orhan D, Ozen S, Besbas N (2015). Anti-interleukin 1 treatment in secondary renal amyloidosis associated with autoinflammatory diseases. Pediatr Rheumatol Online J.

